# Conquering the Nuclear Envelope Barriers by EBV Lytic Replication

**DOI:** 10.3390/v13040702

**Published:** 2021-04-18

**Authors:** Chung-Pei Lee, Mei-Ru Chen

**Affiliations:** 1 School of Nursing, National Taipei University of Nursing and Health Sciences, Taipei 112303, Taiwan; chungpei@ntunhs.edu.tw; 2 Graduate Institute and Department of Microbiology, College of Medicine, National Taiwan University, Taipei 100233, Taiwan

**Keywords:** Epstein–Barr virus, BGLF4 kinase, nuclear egress, BFRF1, nuclear envelope modulation

## Abstract

The nuclear envelope (NE) of eukaryotic cells has a highly structural architecture, comprising double lipid-bilayer membranes, nuclear pore complexes, and an underlying nuclear lamina network. The NE structure is held in place through the membrane-bound LINC (linker of nucleoskeleton and cytoskeleton) complex, spanning the inner and outer nuclear membranes. The NE functions as a barrier between the nucleus and cytoplasm and as a transverse scaffold for various cellular processes. Epstein–Barr virus (EBV) is a human pathogen that infects most of the world’s population and is associated with several well-known malignancies. Within the nucleus, the replicated viral DNA is packaged into capsids, which subsequently egress from the nucleus into the cytoplasm for tegumentation and final envelopment. There is increasing evidence that viral lytic gene expression or replication contributes to the pathogenesis of EBV. Various EBV lytic proteins regulate and modulate the nuclear envelope structure in different ways, especially the viral BGLF4 kinase and the nuclear egress complex BFRF1/BFRF2. From the aspects of nuclear membrane structure, viral components, and fundamental nucleocytoplasmic transport controls, this review summarizes our findings and recently updated information on NE structure modification and NE-related cellular processes mediated by EBV.

## 1. The Structure and Function of the Nuclear Envelope

**Figure 1:**
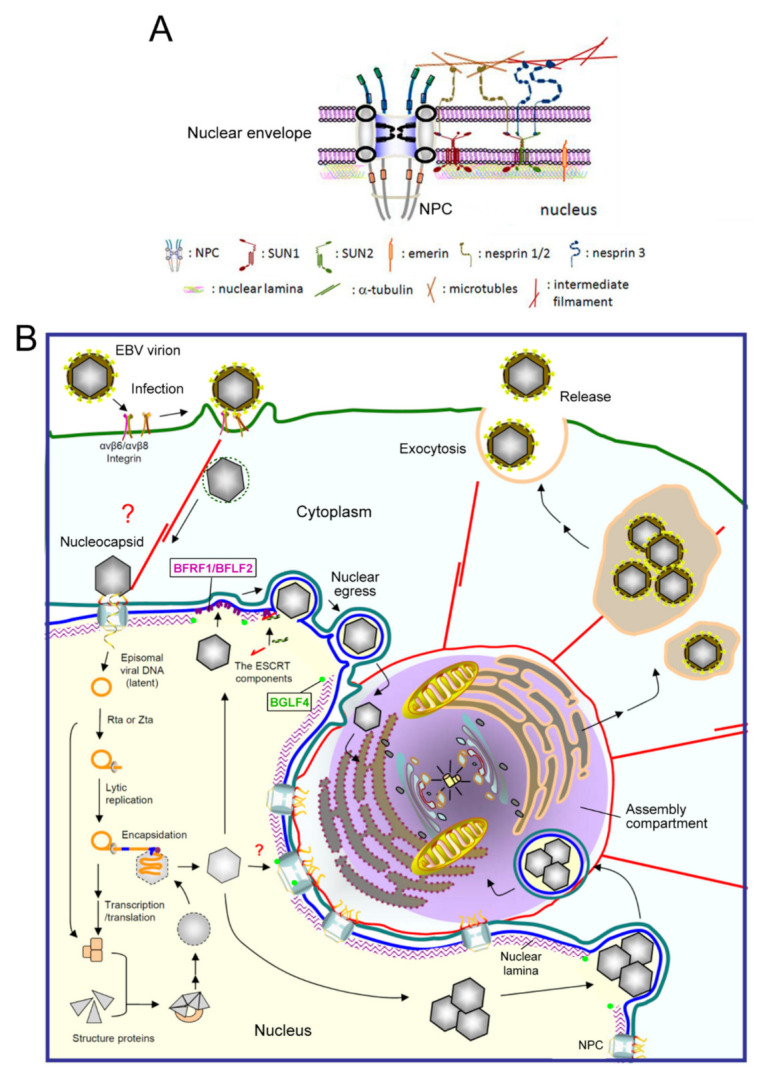
A diagram of EBV virion replication, production, and maturation. (**A**) An overview of the nuclear envelope (NE) structure with selected NE-associated proteins and nuclear pore complexes. The NE is composed of two lipid bilayers, inner and outer nuclear membranes (INM and ONM), and the nuclear lamina formed by lamin filaments. The nuclear pore complexes (NPCs) not only serve as gates for material transport, but also structurally hold the membranes and lamina together. The ONM, which is continuous with the ER, is characterized by cytoskeleton-associated nesprin proteins tethered by SUN1 and SUN2 in the INM. Emerin is an INM integrated protein that anchors on the nuclear lamina. (**B**) After EBV infects cells, the viral genome is injected into the nucleus through nuclear pore complexes. The linear genome is circularized into the episomal form through the terminal repeats and maintained as an episomal form in the latently infected cells. With chemical stimulations or reactivation signals, the viral transactivator Zta or Rta activates replication-related viral proteins and lytic viral DNA replication. Along with the production and assembly of viral capsid proteins, the viral genome is encapsidated into the preformed procapsid to form the mature nucleocapsid. Simultaneously, the viral BGLF4 protein kinase mediates the phosphorylation and partial disassembly of nuclear membrane-underlying nuclear lamina, allowing the access of nucleocapsids to the nuclear membrane. In our observations, the nucleocapsids then traverse through the nuclear envelope with the coordination of the viral nuclear egress complex BFRF1/BFLF2, cellular ESCRT machinery, and Nedd4-like ubiquitin ligases. The cytoplasm-distributed nucleocapsids may subsequently transport into the juxtanuclear “viral assembly compartment”. This specialized compartment, containing highly reorganized membrane structures and organelles, may provide a site for efficient tegumentation and secondary envelopment of the nucleocapsids. Finally, the mature virion is transported close to the cell margins and released from the infected cells by exocytosis. NPC, nuclear pore complex.

In higher eukaryotic cells, the nucleus is surrounded by an envelope composed of the inner and outer nuclear membranes, the perinuclear space between the membranes, and the underlying nuclear lamina network (Figure 1A). These three-layer structures are held in place by the nuclear pore complex (NPC), which functions as a transport gatekeeper for macromolecules trafficking in or out of the nucleus. The outer nuclear membrane is continuous with the rough endoplasmic reticulum. The nuclear lamina is a thin, electron-dense meshwork lining the nucleoplasmic face of the inner nuclear membrane (INM) [1,2] and provides firm structural support for the major components of the nuclear envelope (NE) [3,4]. The integrity of the envelope provides the structural support for nuclear morphology and functions as a platform for coordinating several cellular processes, including DNA repair, cell signaling, migration, and gene expression. The lamina is connected to the inner nuclear membrane through INM proteins (emerin and lamin B receptor), the chromatin proteins (histone H2A/H2B), and also the cytoskeleton-interacting LINC (linker of nucleoskeleton and cytoskeleton) complexes (reviewed in [5]). The physical linkages of LINC are instrumental in the plasticity of cellular organization and are required for processes such as nuclear migration and anchorage, meiotic chromosome movements, centrosome positioning, and the global organization of the cytoskeleton.

NE integrity is essential for cell homeostasis. Alteration of NE structure or components may impact the behavior and the phenotypes of cells in many ways. For decades, deleterious morphological changes of the NE in tumor cells have been recognized as an important parameter for the malignant criteria of tumors [5,6,7]. Many NE-associated diseases, collectively designated nuclear envelopathies or laminopathies, have been identified. Most of these disorders come from mutations in INM proteins or their interacting partners, diseases including muscular dystrophy, lipodystrophy, neuropathy, and progeria (premature aging) syndromes [8,9]. Diseases may be due to mutations or altered expression of alleles, the mechanical stability of the nucleus, or cell cycle regulation. However, the molecular mechanisms that give rise to these diseases and whether pathogen infection may modulate the nuclear structure and contribute to the pathogenesis remain poorly understood [10,11].

## 2. Nuclear Pore Complexes (NPC) Function as Gates and Guardians for Nuclear–Cytoplasmic Transport

The gatekeeper function of the NPC is important for preventing the abnormal distribution of cytoplasmic components or genome-toxic insults to the cellular chromatin. The NPC has a molecular mass of ~125 megaDaltons (MDa) in vertebrates and is composed of multiple copies of a set of 30 diverse proteins, termed nucleoporins (NUPs) [12]. In the middle of the nuclear pore, FG-NUPs, containing Phe-Gly (FG) repeats, play the most relevant role in controlling the nuclear transport receptors (NTR) that shuttle through the NPC. FG-NUPs and NTRs interact through hydrophobic contacts for the translocation step of nuclear transport through the NPC. The canonical transport of most macromolecules (>40 kDa) into the nucleus is controlled by nuclear localization signals (NLS) that are recognized by specific members of the importin receptor family. Proteins containing a bipartite motif or a stretch of basic amino acids bind to an importin alpha and beta heterodimeric receptor complex [13]. During transport, importin α binds to the NLS-bearing protein, and importin β mediates the binding of the transport complex to the FG-NUPs, which subsequently slide through the NPC [14,15]. The small GTPase Ran regulates the association and dissociation of the importin-cargo complex between GTP- and GDP-bound forms [16,17]. For the export of nuclear protein, XPO1 (Exportin-1/Chromosome Region Maintenance 1/CRM1) is the most known mediator. Nuclear export signals (NESs) are short leucine-rich sequences that can be found in many shuttling proteins. Cargo proteins containing NES are recognized by XPO1, which interacts with nucleoporins NUP214 and NUP88 in NPC to transport out of the nucleus in a RanGAP-dependent manner [18,19].

Nuclear transport is also an important step that controls specific signaling pathways. For example, activators of transcription, including IRF3 and NF-κB, are regularly expressed and sequestered in the cytoplasm. They are translocated into the nucleus through phosphorylation-mediated regulation and activate specific innate immune response genes upon stimulation [20,21]. Such regulation could occur in as little as 30 min to activate gene transcription in response to challenge by various pathogens.

## 3. Overview of EBV Lytic Replication

Epstein–Barr virus (EBV) is a human gamma-herpesvirus that infects most of the world’s population. It has a 170–175 kb linear double-stranded DNA genome which encodes approximately 90 open reading frames. The genome is packaged within an icosahedral capsid, approximately 100–120 nm in diameter, surrounded by a proteinaceous tegument and a lipid bilayer envelope. EBV generally establishes two phases in its life cycle, latency and lytic replication [22]. EBV latent proteins help B cell proliferation, including LMP1 (latent membrane protein 1), which can mimic CD40 signaling to activate NF-κB signaling, and EBNA2 (EBV nuclear antigen 2), which can turn on Myc expression. EBV can transform primary B cells into lymphoblastoid cell lines in vitro and may cause post-transplantation lymphoproliferative disease (PTLD). Increasing evidence indicates that lytic phase gene expression also contributes to EBV oncogenesis [23].

EBV mainly infects naïve B lymphocytes and epithelial cells through different receptors, including CR2, HLA class II, and integrins [24]. B cell entry may occur via membrane fusion or endocytosis followed by fusion of the viral membrane with the membrane of the endocytic vesicle [25]. Ephrin receptor A2 was recently identified as the EBV receptor on epithelial cells [24,26,27]. The various steps of EBV replication are illustrated in Figure 1B. After the initial infection, mediated by sequential interactions of viral glycoproteins with host surface receptors, the nucleocapsid is internalized through endocytosis or membrane fusion [28,29,30]. It has been postulated that the nucleocapsid might undergo some structural changes and slide along the microtubule or actin cytoskeleton to the NPC boundary [31]. The linear viral genome is then injected into the nucleus [32]. However, how the EBV genome gets through the NPC and whether viral factors modify the NPC structure to facilitate transport of the viral genome remain unclear. The genome is then circularized and maintained by the EBV encoded nuclear antigen EBNA1 as an episomal form in the nucleus, remaining latent in the host [24].

Upon chemical or stress stimulation, or Ig cross-linking on the surface of B cells, EBV is reactivated and genome replication ensues. Viral lytic genes are expressed in a temporal and sequential order that is divided into three stages, immediate-early (IE), early (E), and late (L). Two immediate-early transactivators, Zta and Rta, are transcribed before viral protein synthesis and can turn on complete viral lytic replication [22]. Most of the early genes are required for viral DNA replication, such as the polymerase processivity factor BMRF1 and CDK1 (cyclin-dependent kinase 1)-like protein kinase BGLF4. The late viral products are mainly structural proteins that are expressed after viral DNA synthesis, such as viral capsid protein VCA and glycoprotein gp350/220 [33]. After DNA replication, the viral genome is packaged into the preformed capsid in the nucleus. With the coordination of cellular machinery and the viral egress complex BFRF1 and BFLF2, the intranuclear nucleocapsids subsequently move towards the inner leaflet of the nuclear membrane and bud from the NE [34,35]. In our unpublished observations, the transported nucleocapsids may obtain their tegument and secondary envelope at the “cytoplasmic assembly compartment”, a specialized cell compartment containing highly reorganized membrane apparatus, cell organelles, and cytoskeletons. Finally, the mature virions containing the cis-Golgi derived membrane are transported and released from the cell through exocytosis [36] (Figure 1B).

## 4. The Nuclear Import of EBV Proteins

To initiate viral DNA replication, Zta and Rta, coordinately bind to the origin of lytic replication (oriLyt) and recruit essential lytic replication proteins to form a core replication complex [37,38]. The viral DNA replication machinery includes the DNA polymerase (BALF5), single-stranded DNA (ssDNA) binding protein (BALF2), DNA polymerase processivity factor (BMRF1), helicase (BBLF4), primase (BSLF1), primase accessory proteins (BBLF2/BBLF3), and uracil DNA glycosylase (BKRF3). Viral DNA replicates in a manner very similar to the mammalian DNA replication system [37,38,39,40]. For oriLyt binding and transactivator function, Zta contains a classical NLS for nuclear targeting. Interestingly, several EBV genes involved in viral DNA replication lack the canonical NLS signals in their coding region. For example, viral DNA polymerase (BALF5) and uracil DNA glycosylase (BKRF3) are transported into the nucleus through interaction with the DNA polymerase processivity factor (BMRF1) [39,41]. Interestingly, the three EBV primase/helicase complex components do not contain canonical NLS but can be imported into the nucleus through interaction with the origin binding protein Zta [42], suggesting EBV evolved with novel pathways for nuclear targeting. In addition, we found the EBV protein kinase BGLF4 enters the nucleus through the direct interaction of its carboxyl-terminal alpha-helical region with the FG-NUP proteins NUP60 and NUP153, independent of importins [43]. BGLF4 also modulates the structure and transport preference of the NPC to facilitate the nuclear import of several EBV lytic proteins, including all three primase/helicase complex proteins and the viral capsid protein BcLF1 [44]. We postulated that the novel nuclear targeting mechanism and BGLF4-mediated NPC preference may interfere with the cellular antiviral response and ensure dominance of the virus for efficient replication.

## 5. The Nuclear Export (Egress) of EBV Nucleocapsids

After genome replication, EBV DNA is packaged into the preassembled procapsid, which primarily consists of the major capsid protein, VCA, and is held together by 320 triplexes formed by two minor capsid proteins, BDLF1 and BORF1, and a small capsid protein, BFRF3, on the hexameric capsomers [45]. The nucleocapsid size is about 100–130 nm, too large to go through the nucleopore. The NE structure is a significant hindrance for the transport of the nucleocapsid into the cytoplasm. Our and others’ studies demonstrated that at least three virus-encoded proteins, including BGLF4 protein kinase and nuclear egress complex BFRF1/BFLF2, modify the NE structure to facilitate the nucleocytoplasmic transport of nucleocapsids.

## 6. EBV BGLF4 Protein Kinase Functions through CDK Mimicry

**Figure 2:**
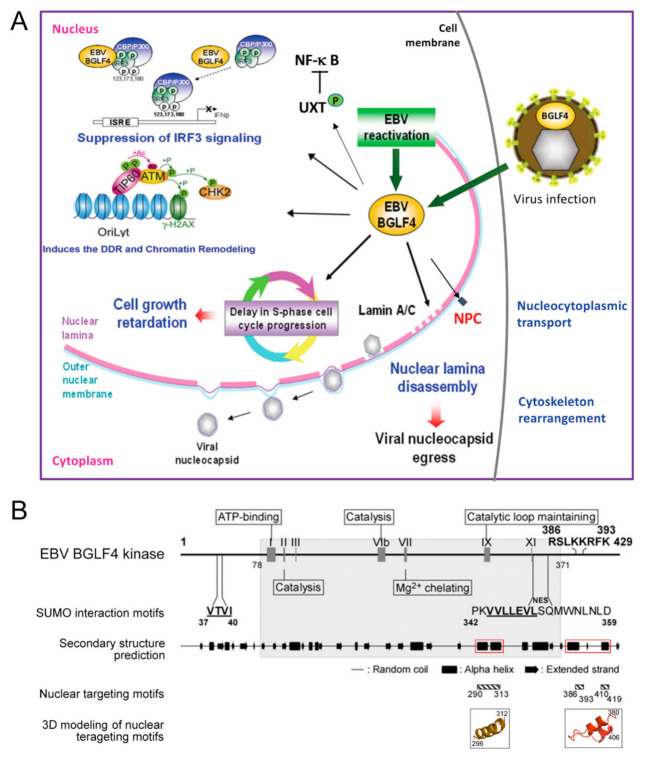
The regulatory functions and the conserved kinase motifs of EBV BGLF4 kinase. (**A**) BGLF4 is a virion-associated protein kinase. After virus infection or reactivation, the mainly nucleus-distributed BGLF4 regulates the cellular environment in many respects. The BGLF4-mediated phosphorylation of IRF3 and UXT is known to suppress the IRF3- and NFκB-mediated cellular suppression of virus replication. BGLF4 regulates the cellular chromatin or chromosome structure by phosphorylating TIP60, condensin, and topoisomerase. The expression of BGLF4 delays cell cycle progression and results in cell growth retardation. These regulations can provide an optimal cellular environment for efficient virus replication. Additionally, BGLF4 plays a crucial role in regulating virion production and maturation. BGLF4 phosphorylates the nuclear lamin A/C and the components of the nuclear pore complex (NPC). The phosphorylation of these structural components attenuates the nuclear envelope barriers and facilitates the transport of viral late proteins and subsequently the nuclear egress of nucleocapsids. In our recent observation, reorganization of the cytoskeleton may also facilitate the maturation and production of the cytoplasm-located nucleocapsids. (**B**) Conserved kinase motifs of BGLF4 were identified by alignment with eukaryotic protein kinases. There are 11 conserved motifs defined in eukaryotic protein kinases [63]. Only motifs I, II, VIb, VII, IX, and XI can be identified in the kinase domain (gray region) of herpesviral kinases [64]. I, ATP binding; II and VIb, catalysis; VII, Mg^2+^ chelating; IX, catalytic loop maintaining. SUMO interaction motifs (SIMs) at a.a. 37–40 and 344–350 and the NES at a.a. 342–359 were identified in a recent study [65]. Amino acids 386–393 were predicted to comprise a putative NLS [66]. However, our study showed that the alpha-helical structure, rather than the positive charges of these amino acids, is critical for BGLF4 nuclear targeting [43]. The putative secondary structure of BGLF4 was analyzed by the GOR secondary structure prediction program (http://www.expasy.ch/tools/ (accessed on 23 May 2012)). Random coils, alpha helices, and extended strands are indicated as thin lines, boxes, and arrows, respectively. The red boxes indicate the BGLF4 unique regions crucial for nucleoporin interaction and nuclear targeting. The hatched boxes underneath the predicted secondary structure indicate the critical regions (a.a. 290–313, 386–393, and 410–419) for BGLF4 nuclear targeting. The critical helical regions for BGLF4 nuclear targeting were also predicted by Protein Structure Prediction Server (PS)^2^ (version 2; http://ps2v2.life.nctu.edu.tw/ (accessed on 23 May 2012)) using casein kinase 2 alpha 1 polypeptide (PDB accession number 3bqcA) as the template and shown in enlarged 3D images for a.a. 296–312 and a.a. 380–406. This figure is adapted and reorganized from our previous study published in Journal of Virology 2012, 86:8072-85 [43].

The EBV BGLF4 protein kinase is a viral tegument protein essential for viral DNA replication and virion maturation [46,47,48,49]. In contrast to alpha-herpes viruses with Us3 and UL13 kinases, EBV encodes the unique BGLF4 kinase during the early through the late stage of virus replication. BGLF4 belongs to be the so-called conserved herpesvirus protein kinases (CHPKs), which recognize Ser/Thr-Proline motifs within their substrates [50]. Herpesviral CHPKs contribute to the initial infection by the virion, the regulation of expression of viral genes, the replication and encapsidation of the viral genome, and the egress and tegumentation of the nucleocapsid [48,50,51,52,53]. Since Ser/Thr-Proline motifs are also targeted by cellular cyclin-dependent kinases (CDKs) and other cell signaling-related kinases, CHPKs are believed to regulate the host cell environment in part through CDK mimicry. BGLF4 kinase phosphorylates multiple cellular substrates to regulate cellular environments for optimal virus replication and production through many processes, including molecules involved in cellular immunity, cell signaling, cell cycle, chromosome structure, nuclear envelope structure, and even cytoskeleton rearrangement [52,53,54], as summarized in Figure 2A. For example, the phosphorylation of BGLF4 on IRF3 and UXT is known to suppress the IRF3- and NFκB-mediated cellular suppression of virus replication [55,56]. BGLF4 regulates the cellular chromatin and chromosome structure by TIP60, condensin, and topoisomerase phosphorylation [51,57]. The expression of BGLF4 delays cell cycle progression at the S phase and results in retardation of cell growth [58]. Additionally, the presence of BGLF4 plays a crucial role in regulating virion production and maturation. The phosphorylation and reorganization of the nuclear lamina and NPC structure by BGLF4 facilitate the nuclear egress of nucleocapsids and viral late protein transportation [44,49]. A panel of viral proteins involved in virus gene regulation, nucleotide metabolism, and virion structure was also identified as BGLF4 substrates by proteomic approaches [51,59]. For instance, BGLF4 interacts with the viral transactivator Zta and regulates its transactivation activity [60]. EBV EBNA1, an essential protein for episomal EBV genome replication and maintenance during latency, is also a substrate and binding partner of BGLF4 [59]. Several reviews have summarized the functions of conserved herpesviral kinases [48,50,61,62]. Of note, a recent study using a quantitative proteomic approach indicates that CHPKs in gamma-herpesviruses have different interaction profiles from those of the α- and β-herpesviruses [53]. In contrast to all herpesviral CHPKs, which modulate cell transcription, replication and the cell cycle, EBV BGLF4 targets preferentially chromatin silencing and DNA replication-associated factors. It is suggested that, even though most CHPKs phosphorylate various cellular factors, the CHPKs of the gamma-herpesviruses have unique characteristics in altering the cellular environment during virus reactivation.

**Figure 3:**
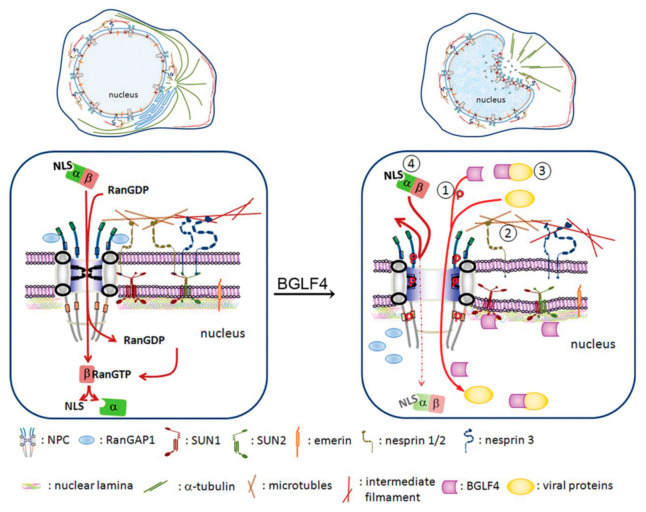
Hypothetical model of the EBV BGLF4-induced structural and functional changes of the nuclear pore complex. (Left part) Under physiological conditions, the nuclear import of NLS-containing proteins is mediated by an importin α-importin β complex and is dependent on RanGTP hydrolysis. (Right part) In the presence of BGLF4, BGLF4 is transported into the nucleus through direct interaction and phosphorylation of FG-NUPs, including NUP62 and NUP153, to dilate the nuclear pores. Simultaneously, BGLF4 also induces reorganization of microtubules and causes changes in the nuclear shape. The nuclear envelope becomes irregular, and the nuclear envelope-associated proteins, such as SUN1 and SUN2, are redistributed. Non-NLS-containing viral proteins can be transported into the nucleus at the same time through at least three different mechanisms: (1) dilated nuclear pores, (2) a microtubule reorganization-dependent mechanism, and (3) direct interaction with or phosphorylation by BGLF4. (4) At the same time, the nuclear import of canonical NLS-containing proteins is partly inhibited. This model is adapted from our previous paper published in Journal of Virology 2015, 89:1703-18 [44].

We previously demonstrated that the expression of BGLF4 alone efficiently modulates chromatin structure and cytoskeleton arrangement, very similar to CDK1-induced promitotic events [57]. Additionally, we found that the ability to induce nuclear lamina disassembly is conserved in all CHPKs [49]. Correspondingly, nuclear retention of viral nucleocapsids was observed in cells infected with a CHPK-deficient recombinant virus or in kinase knockdown experiments [48,67,68,69,70,71]. These observations indicate the contribution of CHPKs to regulating virion maturation and release [49,51,52,53,72,73,74,75]. Besides, we found that BGLF4 targets the nucleus through an unconventional interaction with nucleoporins and is independent of cytosolic factors, unlike other CHPKs [43] (Figure 2B). Through this interaction, BGLF4 modulates the structure and preference of nuclear pore complexes to facilitate the nuclear import of EBV lytic proteins [44] (Figure 3). A recent study reported that the cellular CDK1-substrate SAMHD1 (sterile alpha motif and HD) is also regulated by BGLF4 and other CHPKs [76]. The phosphorylation of SAMHD1 leads to an increased cellular dNTP pool and therefore facilitates virus replication. Overall, these observations suggest that the CHPKs-mediated modulation of nuclear structure or the cellular environment is critical for overcoming the natural barrier or cellular limitation during herpesviral virion replication and maturation. For the nuclear egress process, BGLF4 plays an important role in phosphorylating nuclear lamin A/C and causes partial disassembly of the nuclear lamina. Therefore, the relaxed lamina structure facilitates the remodeling of nuclear membranes and subsequent recruitment of ESCRT components to transport nucleocapsids.

## 7. EBV Nuclear Egress Complex (NEC): BFRF1 and BFLF2 Proteins

The study regarding the nuclear egress of herpesviruses was pioneered by Roller and Baines’ Labs that made the first and critical observations of how viruses encode these nuclear egress functions [77]. Based on the “envelopment–de-envelopment–re-envelopment” model, the large herpesviral nucleocapsids (100–130 nm) begin budding through the nuclear membrane via a transient envelopment. This is mediated first by the viral protein kinase and nuclear membrane-associated proteins at the inner nuclear membrane (INM), with the local disassembly of compact nuclear lamina for primary envelopment. The nucleocapsids with the primary envelope may then de-envelope by fusing with the outer nuclear membrane (ONM), be transported from the perinuclear space to rough endoplasmic reticulum cisternae or, alternatively, released from the disintegrated nuclear envelope [78,79]. After release from the NE-derived structures, the nucleocapsids subsequently become associated with viral tegument proteins and glycoproteins at cytoplasmic apparatuses for the final maturation of virions [34,80,81,82].

In herpesviruses, the conserved homologs of UL34 and UL31 are believed to be the major viral components that mediate the primary envelopment. The homologs of herpes simplex type 1 (HSV-1) UL34 are type II integral membrane proteins, which localize predominantly to the ONM, INM, and ER, whereas the UL31 homologs are nuclear matrix-associated phosphoproteins [83,84]. Transient overexpression of the UL34/UL31 homologs of HSV-1 or human cytomegalovirus (HCMV) induces subtle alterations of the nuclear lamina [85,86], suggesting the homologs of UL34 and UL31 regulate potentially the structure of the nuclear membrane. More recently, the NE modulation of the UL34/UL31 complexes, also called nuclear egress complexes (NECs), has received much attention among herpesviruses [35,81,87,88,89,90]. Using various super-resolution imaging techniques and biophysical analysis, the HCMV and HSV-1 heterodimeric core NECs were shown to assemble and form hexameric latticed structures [87,91]. NECs contribute to recruiting effectors and reorganizing the NE, allowing the docking and nuclear egress of viral nucleocapsids [81,87,88,89,90,91,92,93]. So far, the UL34 homologs are believed to be rate-limiting factors during the nuclear egress among the NEC components, such as pUL34 in HSV-1 [94], pUL50 in HCMV [95], and BFRF1 in EBV [84]. Intriguingly, even though herpesviral NECs all show a similar structural basis in their conserved regions, some unique activities are observed in individual herpesviruses.

The EBV gene products of BFRF1 and BFLF2, the positional homologs of UL34 and UL31 in HSV-1, have been shown to regulate the primary egress of nucleocapsids [96]. Coexpression of BFRF1 and BFLF2 induces modification of the nuclear membrane, which is frequently observed in cells with EBV replication [97,98]. Expression of BFRF1 in 293 cells induces the stacking of a multilayered nuclear membrane and vesicle-like structures [84,99]. Coexpression of BFRF1 and BFLF2 dramatically reorganizes the multilayered nuclear membrane and induces the dilated packaged cisternae and even concentric whorls [96]. Similar structural changes of nuclear envelope were also observed in cells expressing the homologous NEC proteins in other herpesviruses [100,101]. Consistently, the amounts of intracytoplasmic nucleocapsids are reduced in mutant EBV lacking BFRF1 or BFLF2 [84,102]. These observations indicate crucial roles of BFRF1 and BFLF2 in inducing the change of the nuclear membrane for efficient primary envelopment.

In contrast to BFRF1, knowledge regarding the function of EBV BFLF2 is limited. Deletion of the BFLF2 gene from an EBV bacmid induced the accumulation of empty capsids in the nucleus and reduced virion release, suggesting BFLF2 is required for correct DNA packaging and the nuclear egress of viral nucleocapsids [102,103]. When expressed alone, BFLF2 is distributed mainly in the nucleus. However, it colocalizes with BFRF1 at the nuclear rim and in NE-derived vesicles in coexpressing cells, suggesting that interaction between BFLF2 and BFRF1 is critical for their proper function [103]. Unlike its herpesviral homologs with a conserved nuclear localization signal (NLS), noncontinuous alkaline and histidine residues in BFLF2 function together as a noncanonical NLS for its nuclear targeting, in an importin β-dependent manner [103,104]. Interestingly, in a high-throughput yeast two-hybrid screening system, BFLF2 protein was highly connected with various human and EBV proteins, resembling a hub in the interactome network [105], suggesting that BFLF2 may function as a scaffold for nuclear cargo transport or cell signaling. Therefore, studying the regulatory function of BFLF2 may provide an insight into virus-host interactions and changes in the nuclear environment during EBV reactivation.

## 8. Cellular ESCRT Machinery and Ubiquitination Regulate the Nuclear Egress of EBV

The endosomal sorting complex required for transport (ESCRT) machinery is evolutionarily conserved and involved in catalyzing the scission of membrane necks in autophagy, cytokinesis, endosome sorting, biogenesis of multivesicular bodies (MVBs), NE reformation and repair, and the maturation and release of enveloped virions. In contrast to the cellular membrane-scission protein dynamin family, which cleaves membrane necks by constricting them from the outside, membrane scission mediated by the ESCRT machinery is from inside the neck [106,107]. The ESCRT components (also known as class E proteins) comprise five multiprotein complexes, ESCRT-0, -I, -II, -III, Vps4 (vacuolar protein sorting-4) ATPase, and several ESCRT-associated proteins [108,109]. ESCRT-0, -I, and -II are soluble complexes that shuttle between cytosolic and membrane-bound forms. These components coordinate together sequentially to curve the membrane and recruit ESCRT-III for scission of the membrane neck. ESCRT-III protein CHMPs (charged multivesicular body proteins) are soluble monomers that assemble on membranes to form tight filamentary spirals and are released from the membranes at the final stage, with other ESCRT proteins, by a transient ATP-dependent reaction of Vps4. In addition to the regular composition, cellular ESCRT-I protein TSG101 (tumor susceptibility gene 101) alternatively activates the spiral assembly of ESCRT-III through bridging by the ESCRT associated-protein, apoptosis linked gene-2 interacting protein X (Alix) [110].

Accumulating studies have reported that components of the ESCRT machinery are used by many enveloped viruses for budding and release from cells. For example, the dynamics of ESCRT protein recruitment in retroviruses were found to be extremely transient (~1–3 min) and sufficient for their functions on the cytoplasmic membrane for virus release [111]. In contrast to enveloped RNA viruses, the contribution of ESCRT machinery to the maturation of enveloped herpesvirus has emerged only in the past decade. For instance, the contribution of ESCRT to virion release and cytoplasmic re-envelopment of HSV-1 and HCMV has been characterized using dominant-negative (DN) inhibitors of ESCRT and siRNA strategies [112,113,114,115]. By immunofluorescence analysis, the cytoplasmic nucleocapsids and envelope components are associated with MVB and colocalized with endosomal markers in infected cells [116,117]. As observed by electron microscopy (EM), HHV-6 also induces MVB formation and cytoplasmic egress through an exosomal release pathway [118], suggesting that herpesviruses use the ESCRT machinery for their membrane-dependent maturation in the cytoplasm.

**Figure 4:**
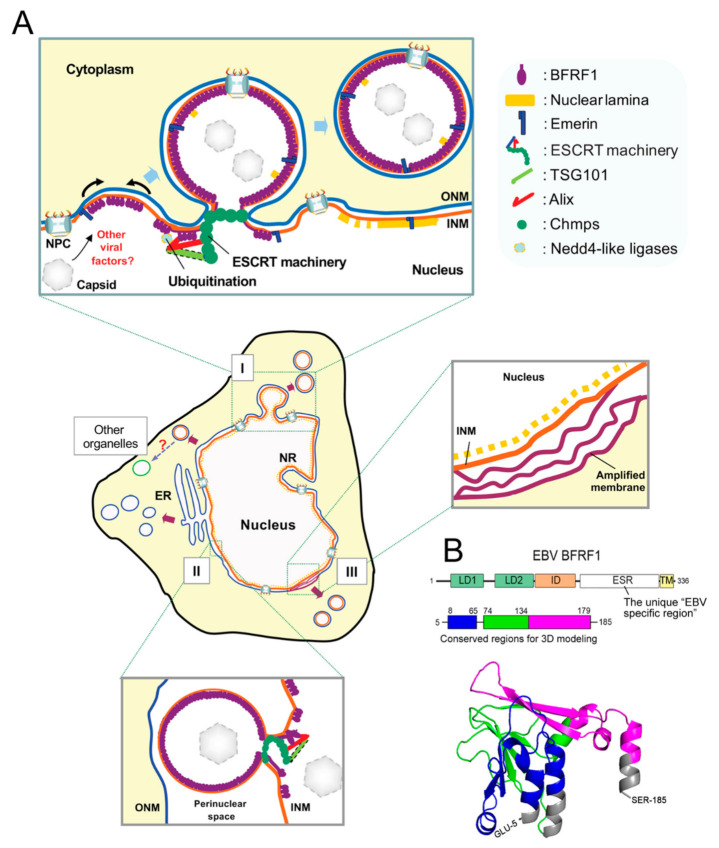
A hypothetical model of the interaction among BFRF1, cellular ESCRT components, and Nedd4-like ligases in vesicle formation and modulation of the nuclear membrane. (**A**) After EBV reactivation, membrane anchoring BFRF1 targets to the nucleus-associated membranes. BFRF1 potentially regulates the nuclear membrane through protein oligomerization. It then recruits Alix, Nedd4-like ubiquitin ligases, and, sequentially, other ESCRT components to generate vesicles derived from the double layer nuclear envelope (I), outer nuclear membrane (ONM), or from the inner nuclear membrane (INM) (II). These nuclear membrane-derived vesicles may contain some nucleoporins and other INM proteins, such as lamin A/C or emerin. Cooperating with other viral factors, such as BFLF2, the BFRF1-mediated vesicles may further recruit and pack some nucleocapsids for the subsequent nuclear egress. In addition, the expression of BFRF1 induces the formation of multilayered cisternal nuclear membranes (III), which may provide a more membranous structure for viral budding. ER, endoplasmic reticulum. NPC, nuclear pore complex. This model represents a refined version of BFRF1-ESCRT component interactions as analogously published in PLOS Pathogens 2012, e1002904 [99]. (**B**) Prediction of the 3D structural BFRF1, based on the HCMV 5DOB chain B. The EBV-specific region (ESR), consisting of amino acids 180–313, could not be predicted because of a lack of sequence similarity. The LD1 domain is shown in green, the LD2 domain is shown in blue, and the ID domain is shown in pink. The regions involved in BFRF1 interaction are indicated in the BFRF1 functional domains and in the 3D modeling. This figure is adapted and reorganized from a previous figure published in Journal of Virology 2020, 94: e01498-19 [103].

In EM images of EBV-infected cells, we observed that virus reactivation induces a vesicle-like ultrastructure [119,120]. Intriguingly, the expression of dominant-negative ESCRT proteins caused a blockade of virion release and retention of the viral NEC protein BFRF1 at the NE [99]. BFRF1 can interact with the adaptor protein Alix, recruit ESCRT components, and mediate vesicle formation from the nucleus-associated membrane. Remarkably, inhibition of the ESCRT machinery by dominant-negative components abolished BFRF1-induced vesicle formation, leading to the accumulation of viral DNA and capsid proteins in the nuclei of EBV-replicating cells. This observation suggests that the ESCRT machinery plays a crucial role during the nuclear egress of EBV. As revealed in Figure 4A, the membrane anchoring BFRF1 potentially oligomerizes and recruits Alix, Nedd4-like ubiquitin ligases and, sequentially, other ESCRT components to generate the vesicles from the nucleus-associated membrane after EBV reactivation. Cooperating with other viral factors, such as BFLF2, the BFRF1-mediated vesicles may further recruit and pack nucleocapsids for their subsequent nuclear egress. Even though the topological regulation of Alix in the process remains unclear, the EBV uses an alternative vesicular nucleocytoplasmic transport for its nuclear egress, other than the postulated envelopment–de-envelopment model proposed for alpha- and beta-herpes viruses. In addition to the ESCRT components, we further demonstrated that the membrane modulating functions of BFRF1 are regulated by ubiquitination [119]. Elimination of possible ubiquitination on BFRF1 hampers NE-derived vesicle formation and virus maturation. The Nedd4-like ubiquitin ligase Itch is preferentially associated with Alix and BFRF1, and required for BFRF1-induced vesicle formation. As BFRF1 enforces the dynamics of the NE and promotes the transfer of viral nuclear cargos, EBV BFRF1 may serve as a potent molecule to explore the vesicular nucleocytoplasmic transport pathway in higher eukaryotes [120]. Further investigation regarding how cellular Alix contributes topologically to this NE modulation may also provide a new understating of the dynamics and fundamental regulations of the NE.

## 9. The Unique Sequences and Features of EBV NEC Proteins

Through protein alignments with herpesviral homologs, two putative L-like motifs (LDs, ^62^YKFL^65^, and ^74^YPSSP^78^), but not conventional L motifs (PTAP, PPXY, and YXXL), an EBV-specific (ESR, a.a. 180–313), and a transmembrane region (TM, a.a. 314–336) were identified within the amino-terminus of EBV BFRF1 ([99] and unpublished data]) (Figure 4B). So far, the EBV-specific region is known to be critical for several BFRF1-mediated functions, such as vesicle formation, Itch-Alix complex, and BFLF2 interaction [99,103,119]. In contrast, EBV BFLF2 shares higher protein similarities with other homologs than does BFRF1 [103]. Intriguingly, the nuclear trafficking and critical residues responsible for regulating partner protein interaction and function are different from its UL31 homologs [103,104]. This suggests that EBV NEC proteins may have unique features different from those of herpesviral homologs. A recent study based on high-resolution protein crystal structures indicates that the γ-herpesviral EBV NEC shares some common folds with α- and β-herpesviruses; however, the primary amino acid sequences in herpesviral core NEC proteins are diverse. The structurally conserved regions are located mainly in the N-terminal regions of EBV BFRF1 (a.a. 2–192) and BFLF2 (a.a 78–110). Notably, both EBV BFRF1 and BFLF2 display several structural particularities [121]. Correspondingly, even though BFRF1 potentially mediates the phosphorylation and redistribution of nuclear emerin, similar to other herpesviral homologs [122], only EBV BFRF1 potently modulates nucleus-associated membranes and induces cytoplasmic vesicles independently of BFLF2 [99,123]. Altogether, these observations suggest that the core NEC of EBV accounts for its unique activities, in addition to the common function in regulating the nuclear structure and protein- or membrane-rearranging functions.

After nuclear egress, the nucleocapsids in the vesicles may be transported through the ER to Golgi pathways and obtain the final envelope with tegument proteins for morphogenesis [124]. Our current study also suggests the endoplasmic reticulum Golgi intermediate compartment (ERGIC), the origin of intracellular membrane genesis between ER and Golgi, may also be involved in the formation of viral cytoplasmic assembly compartment (manuscript in preparation)

## 10. Is the Vesicular Nucleocytoplasmic Transport Pathway Also Present in the Absence of Virus Infection?

As described in the first part of this review, the nuclear pore-mediated nucleocytoplasmic transport controls in the cell need to be tightly regulated. We may wonder whether EBV utilizes a pre-existed cellular pathway for its nuclear egress. Some studies in the past decade have suggested that an NPC-independent vesicular nucleocytoplasmic transport system is conserved in eukaryotes. For example, in response to nutrient starvation, *Saccharomyces* buds double-membrane vesicles (piecemeal microautophagy or late nucleophagy) from the NE into the cytoplasmic vacuole for selective degradation of non-essential nuclear components, such as portions of the NE [125,126]. Studies in *Drosophila* also indicate that large ribonucleoprotein particles are transported across the NE by a vesicle-mediated export for protein synthesis [127,128]. An unconventional export mechanism was also reported to mediate the selective degradation of nuclear lamin or emerin in the cytoplasm of mammalian cells [129,130]. These studies suggest that an NPC-independent vesicular nucleocytoplasmic transport system appears to be conserved among eukaryotes and may cooperate with cytoplasmic processes, such as autophagic proteolysis, for the hemostasis of nuclear components.

## 11. EBV Putatively Hijacks the Endogenous Transporting/Clearance Pathway for Its Maturation

Autophagy is an important mechanism for removing cytoplasmic protein aggregates and damaged organelles, especially when the cellular chaperone capacity or the ubiquitin-proteasome system is overwhelmed or ineffective. Several cellular factors, such as p62 (SQSTM1), ALFY (autophagy-linked FYVE protein), and yeast Atg8 homolog LC3s or GABARAPs, have been shown to be crucial for the selective clearance of cytoplasmic aggresomes [131,132]. Starting with the binding of ubiquitinated substrates or cargos by the ubiquitin-binding receptor p62, the adaptor molecule ALFY binds sequentially to membrane-anchored GABARAP and induces autophagosome formation [133,134,135]. Autophagosomes may then fuse with endosomes to form amphisomes and, finally, fuse with lysosomes for autophagic proteolysis [34,136]. So far, autophagy dysregulation has been linked to several human diseases, such as autoimmune diseases, protein aggregation diseases, and cancers [137]. Herpesviruses, such as EBV and KSHV, also use various strategies to modulate the autophagy pathway for their replication and to overcome the immune response [137,138].

**Figure 5:**
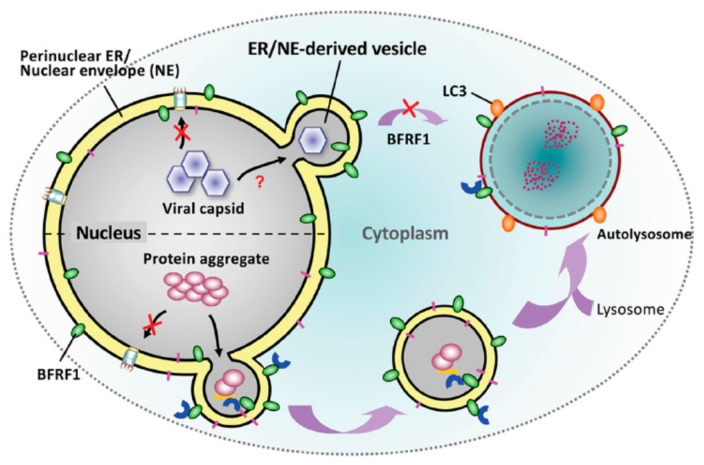
The hypothetical model for the cellular and viral regulation of EBV BFRF1-induced nuclear envelope (NE) modulation. The NE plays a pivotal role in cellular signaling and homeostasis. During EBV reactivation, the viral NE protein BFRF1 can modulate NE structure and form cytoplasmic vesicles by recruiting cellular ESCRT components and Itch ubiquitin ligase [99,119]. The ER/NE-derived vesicles provide nucleocytoplasmic transport for the nuclear egress of nucleocapsids (upper part). The presence of BFRF1 may mediate the blockage of late autophagic proteolysis for transported nucleocapsids in the cytoplasm [140]. Previously, we showed that BFRF1-induced vesicles potentially mediate the delivery and clearance of nuclear protein aggregates through autophagy proteolysis in the cytoplasm [120] (lower part). This information obtained may provide insights into the fundamental functions of the NE and knowledge of NE-related and protein aggregation diseases.

In EBV, the presence of BFRF1 was known to promote early autophagic steps, which is essential for EBV reactivation, and mediate the blockage of late autophagic steps [139,140]. We previously demonstrated that BFRF1 protein recruits cellular ESCRT components to modulate the NE for the nuclear egress of EBV [99]. Based on the characteristic of BFRF1 protein in driving membrane dynamics, we explored the possibility that elicitation of NE-derived vesicles by ectopic BFRF1 expression may improve the translocation and clearance of nucleus-located protein deposition [120]. We found BFRF1-induced vesicles fuse putatively with autophagic vacuoles in the cytoplasm. ALFY-mediated autophagic proteolysis subsequently leads to the clearance of nuclear aggregates (Figure 5). Therefore, it is suggested that viral nucleocapsids may be recognized as similar to the protein deposits in the cellular environment. EBV putatively employs or hijacks parts of the endogenous transporting/clearance pathway, originally responsible for protein aggregate catabolism, for nucleocapsid nucleus egress.

Correspondingly, some studies indicate that EBV uses autophagic machinery for transport and cytosolic maturation [139,141]. The presence of EBV BFRF1 is crucial for the early steps of autophagy and blocks the final proteolysis in the EBV life cycle [140]. These observations suggest that BFRF1-induced NE vesicles may provide the autophagic vehicles for cytoplasmic transport or the membrane source for cytoplasmic re-envelopment of EBV, and mediate virus escape from lysosomal proteolysis for efficient virion production. If that is the case, it will be worthwhile to investigate further how BFRF1 prevents the cytoplasmic fusion of lysosomes during virus maturation. On the other hand, recurrent EBV reactivation is known to enhance the genome instability and tumorigenicity of nasopharyngeal carcinoma [142]. If the presence of EBV BFRF1 improves the dynamics and trafficking of intracellular vesicles, it would be interesting to investigate whether repeated virus reactivation interferes with the regular transport pathway and contributes to the cytopathogenesis of cancer cells.

## 12. Coda: Is the Nucleocytoplasmic Pathway a New Target for Protein Aggregation Diseases?

Intracellular protein aggregates are a pathological hallmark of several neurodegenerative diseases that impact the global population today. Among the protein aggregation diseases, the polyglutamine (polyQ) expansion disorders, such as Huntington’s disease, spinobular muscular atrophy, and the spinocerebellar ataxia 1, 3, and 7, involve cytotoxic, nonnative, aggregation-prone conformations because of the presence of long homopolymeric tracts of glutamine. So far, most strategies for eliminating protein aggregates have focused on reducing gene expression of the pathogenic proteins by RNA-targeting, reducing aggregate formation by pharmacologic or molecular chaperone treatment, or facilitating protein degradation by the ubiquitin–proteasome system [143,144]. Nevertheless, recent studies also indicated that autophagy or lysosome enhancer is a potential protein reduction strategy. For example, treatment of autophagy enhancer improves the clearance of proteolipid aggregates, reduces neuropathology, and prolongs survival of diseased mice [145]. This suggests that autophagy is a powerful system for clearing cell protein deposits. Since the unique EBV BFRF1 stimulates the protrusion of the NE-associated membrane for aggregated protein and links potentially to cytoplasmic autophagy, a similar vesicular nucleocytoplasmic transport across the intact membrane may serve as an ideal pathway for eliminating protein aggregates. Our idea was to test whether the protein deposits can be engulfed by the NE membrane, moved, and subjected to the cytoplasmic degradation system.

Using a system mimicking natural cell conditions in neurodegenerative diseases, we previously found that eliciting vesicular transport by ectopic BFRF1 expression reduces the accumulation of nuclear aggregate in neuroblastoma cells [120]. Thus, it suggests that activation of cellular component translocation by BFRF1 can be exploited to clear pathogenic protein aggregates from neuronal cells. It would be interesting and worthwhile to investigate further whether improving membrane dynamics using EBV BFRF1 can be adapted to an organismal level, such as transgenic animal systems, for the elimination of protein deposits. Nevertheless, the viral origin of BFRF1 may be a concern for its direct use in vivo. Further studies to characterize the safety of driving vesicular nucleocytoplasmic transport in vivo and explore the endogenous cellular factors or small molecule components capable of activating intrinsic vesicular transport are likely to be helpful for the therapy of protein aggregation diseases.
