# Correction: Lee, C.-P.; Chen, M.-R. Conquering the Nuclear Envelope Barriers by EBV Lytic Replication. *Viruses* 2021, *13*, 702

**DOI:** 10.3390/v15101991

**Published:** 2023-09-25

**Authors:** Chung-Pei Lee, Mei-Ru Chen

**Affiliations:** 1 School of Nursing, National Taipei University of Nursing and Health Sciences, Taipei 112303, Taiwan; chungpei@ntunhs.edu.tw; 2 Graduate Institute and Department of Microbiology, College of Medicine, National Taiwan University, Taipei 100233, Taiwan

In the original publication [1], the figures were arranged at the end of the manuscript with the references. After the figures were moved and merged into the text, the references were rearranged. Somehow, the web version is a combination of the correct text and the old reference section. 

The original References [142–145] should be moved after Reference [62], listed as References [63–66]. With this correction, the order of some references has been adjusted accordingly. The authors state that the scientific conclusions are unaffected. This correction was approved by the Academic Editor. The original publication has also been updated.
